# Do Emotion Regulation Abilities in the Lab and in Daily Life Map Onto Each Other for Older Adults?

**DOI:** 10.1093/geroni/igaf122.049

**Published:** 2025-12-31

**Authors:** Jocelyn Lai, Tabea Springstein, Claire Growney, Tammy English

**Affiliations:** Washington University in St. Louis, St. Louis, Missouri, United States; University of California, Riverside, Riverside, California, United States; Stanford University, Stanford, California, United States; Washington University in St. Louis, St. Louis, Missouri, United States

## Abstract

Despite natural decline in physical and cognitive health, older adults experience greater emotional well-being through prioritization and selective allocation of resources towards socioemotional goals. As such, older adults are theorized to have greater emotion regulation (ER) abilities. Empirical findings, however, are mixed regarding age-related strengths in ER. Laboratory-based assessments typically elicit higher-arousal negative affect, a context that older adults experience less of in daily life and may require more cognitive effort. Thus, ER success assessed in these types of experimental paradigms may not be reflective ER success as assessed in daily life for older adults with or without mild cognitive impairment (MCI) compared to younger adults. 211 adults (n = 66 younger adults; n = 87 cognitively normal [CN] older adults; n = 58 older adults with mild cognitive impairment [MCI]) completed a lab-based paradigm of ER and ecological momentary assessments of ER (7x for 9 days). Overall, ER success in the laboratory was minimally associated with ER success in daily life across all three groups. There were, however, age-related advantages in daily ER success, even among older adults with MCI. Our findings suggest that laboratory assessments may capture distinct aspects of ER ability compared to daily life assessments. Future work should test modifications to lab-based assessments to better capture how older adults engage in ER in daily life and consider other factors that may strengthen or weaken the connection between laboratory and in daily life.

